# Cultured lymphocytes’ mitochondrial genome integrity is not altered by cladribine

**DOI:** 10.1093/cei/uxad112

**Published:** 2023-10-20

**Authors:** Elina Järvinen, Fumi Suomi, James B Stewart, Dimitri Guala, Miko Valori, Lilja Jansson, Janne Nieminen, Thomas G McWilliams, Pentti J Tienari

**Affiliations:** Merck OY, Espoo, Finland (an affiliate of Merck KGaA, Darmstadt, Germany); Translational Stem Cell Biology and Metabolism Program, Research Program Unit, Faculty of Medicine, University of Helsinki, Helsinki, Finland; Max Planck Institute for Biology of Ageing, Cologne, Germany; Wellcome Centre for Mitochondrial Research, Biosciences Institute, Faculty of Medical Sciences Newcastle University, Newcastle Upon Tyne, UK; Merck AB, Solna, Sweden (an affiliate of Merck KGaA, Darmstadt, Germany); Department of Biochemistry and Biophysics, Stockholm University, Stockholm, Sweden; Translational Immunology Research Program, University of Helsinki, Helsinki, Finland; Translational Immunology Research Program, University of Helsinki, Helsinki, Finland; Helsinki University Hospital, Neurocenter, Helsinki, Finland; Translational Immunology Research Program, University of Helsinki, Helsinki, Finland; Helsinki University Hospital, Neurocenter, Helsinki, Finland; Translational Stem Cell Biology and Metabolism Program, Research Program Unit, Faculty of Medicine, University of Helsinki, Helsinki, Finland; Department of Anatomy, Faculty of Medicine, University of Helsinki, Helsinki, Finland; Translational Immunology Research Program, University of Helsinki, Helsinki, Finland; Helsinki University Hospital, Neurocenter, Helsinki, Finland

**Keywords:** cladribine, multiple sclerosis, mitochondrial DNA, lymphocytes

## Abstract

Cladribine tablets are a treatment for multiple sclerosis with effects on lymphocytes, yet its mode of action has not been fully established. Here, we analyzed the effects of cladribine on mitochondrial DNA integrity in lymphocytes. We treated cultured human T-cell lines (CCRF-CEM and Jurkat) with varying concentrations of cladribine to mimic the slow cell depletion observed in treated patients. The CCRF-CEM was more susceptible to cladribine than Jurkat cells. In both cells, mitochondrial protein synthesis, mitochondrial DNA copy number, and mitochondrial cytochrome-*c* oxidase-I mRNA mutagenesis was not affected by cladribine, while caspase-3 cleavage was detected in Jurkat cells at 100 nM concentration. Cladribine treatment at concentrations up to 10 nM in CCRF-CEM and 100 nM in Jurkat cells did not induce significant increase in mitochondrial DNA mutations. Peripheral blood mononuclear cells from eight multiple sclerosis patients and four controls were cultured with or without an effective dose of cladribine (5 nM). However, we did not find any differences in mitochondrial DNA somatic mutations in lymphocyte subpopulations (CD4+, CD8+, and CD19+) between treated versus nontreated cells. The overall mutation rate was similar in patients and controls. When different lymphocyte subpopulations were compared, greater mitochondrial DNA mutation levels were detected in CD8+ (*P* = 0.014) and CD4+ (*P* = 0.038) as compared to CD19+ cells, these differences were independent of cladribine treatment. We conclude that T cells have more detectable mitochondrial DNA mutations than B cells, and cladribine has no detectable mutagenic effect on lymphocyte mitochondrial genome nor does it impair mitochondrial function in human T-cell lines.

## Teaser

Cultured leukemic human T-cell lines and cultured *ex vivo* human peripheral mononuclear cells from patients with multiple sclerosis and controls were treated with cladribine *in vitro* . In the T-cell lines mitochondrial protein synthesis, DNA copy number and mutagenesis were not affected by cladribine. In the *ex vivo* lymphocyte subpopulations (CD4+, CD8+, and CD19+), there were no significant differences in mitochondrial DNA mutations between treated versus nontreated cells.

## Graphical Abstract

**Graphical Abstract:**
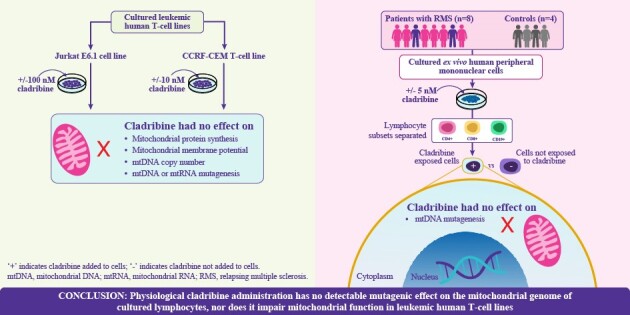

## Introduction

Multiple sclerosis (MS) is a chronic inflammatory disease of the central nervous system. Epstein-Barr virus (EBV) infection has recently been identified as the outstanding risk factor for MS [1]. Dysregulated B- and T-lymphocyte EBV responses as well as microglial activation are considered to underlie the mechanisms of MS [2–4]. Cladribine tablets, an oral formulation of cladribine, is indicated for highly active relapsing MS in the European Union and for relapsing and active secondary progressive disease in the United States [5, 6]. Human lymphocytes, the key cell types in MS pathophysiology, are most vulnerable to cladribine [7, 8]. In addition, inhibitory effect on activated microglia has been reported [9].

Cladribine (2-chloro-deoxyadenosine, 2-CdA), a nucleoside analog of deoxyadenosine, is resistant to adenosine deaminase (ADA), allowing its accumulation in cells [10]. Phosphorylation of cladribine to its active triphosphate form (2-CdATP) is regulated by enzymes of the deoxyribose nucleotide salvage pathway, required for DNA synthesis [10]. The intracellular accumulation of 2-CdATP impairs both nucleic acid synthesis and repair [11, 12]. Cladribine also inhibits ribonucleotide reductase, which regulates deoxyribonucleotide synthesis *via* the *de novo* nucleotide pathway [13]. These combined effects are predicted to induce apoptotic cell death [11, 14, 15].

Lymphocytes require *de novo* and salvage nucleotide pathways to sustain DNA replication requirements that characterize clonal expansion and development. To regulate nucleotide concentration, lymphocytes express high levels of ADA which normally restricts deoxynucleotide cytotoxicity *via* deamination of adenosines to inosines [10]. Although the plasma half-life of cladribine is less than 24 hours [16], lymphocyte depletion *in vivo* is a process that occurs over months (median nadir at 2 months) [7]. The effects of cladribine on lymphocyte depletion are dose dependent, with high doses inducing acute cytotoxicity [17], while the doses used to treat MS induce slow depletion of lymphocytes. The precise mode of action arising from therapeutic cladribine administration is not fully understood in MS, as the temporal kinetics of depletion are protracted with effects on both resting and dividing cells [7].

Cladribine administration has been proposed to induce mitochondrial dysfunction in cultured human leukemic cells, and effects on a range of phenotypes have been reported at high doses [14, 15]. It has also been previously shown that other nucleoside analogs are associated with mitochondrial toxicity [18]. Mitochondria are dynamic signaling organelles harboring a small multicopy genome (mtDNA) that encodes essential subunits of the oxidative phosphorylation machinery. Although replication and transcription of mtDNA occurs independently of the cell cycle, it depends upon the nucleotide salvage pathway [19]. Thus, cladribine could damage the mitochondrial genome by altering nucleotide metabolism. However, the effects of lower cladribine concentrations, mimicking treatment of MS, on mitochondrial integrity have not been investigated. Here, we studied whether cladribine impairs mitochondrial gene expression and mtDNA composition in cultured leukemic human T-cell lines and primary human lymphocytes isolated from patients with MS and healthy controls at such lower cladribine concentrations.

## Materials and methods

### Cell lines and primary cells

The human leukemic Jurkat E6.1 cell line (pseudodiploid, modal No 46) and the CCRF-CEM (ECACC 85112105) cell line (2*n* = 46) were obtained from the European Collection of Authenticated Cell Cultures (ECACC) general cell collection and cultured according to the recommended instructions (Jurkat E6.1: RPMI 1640; 2 mM glutamine; 10% FBS) (CCRF-CEM: RPMI 1640; 2 mM glutamine; 20% FBS). Dose response curves were established in the Jurkat E6.1 and CCRF-CEM cell lines using doses in the range of cladribine plasma concentrations in patients treated with cladribine tablets [5, 20]. Extended culturing of cell lines involved fresh media replacement (including cladribine) every 2 days to maintain cells at the recommended growth density.

### *Ex vivo* sample collection from MS patients and controls

Participants for giving blood samples were recruited at the Department of Neurology of Helsinki University Hospital. This study has been approved by the regional ethics committee at the Helsinki University Hospital (HUS/2935/2018). Each participant gave informed consent for blood sampling for this research. The MS patients fulfilled the McDonald 2001 criteria for relapsing MS [21], and all had oligoclonal bands or elevated IgG index in the diagnostic cerebrospinal fluid examination. None of the patients were using disease-modifying treatment at the time of sampling or had been on a treatment with a long-lasting impact on the immune response. The mean age of the MS patients was 44 years (range 31–56 years), the mean duration of disease after diagnosis was 9.4 years (range 3–20 years), and 75% were females. The controls were healthy volunteers with a mean age of 50 years (range 41–57 years), 50% were females. The demographic and clinical characteristics of the participants are shown in Supplementary Table S1.

### *Ex vivo* human peripheral blood mononuclear cell cultures

120 mL of human venous blood was harvested in lithium heparin tubes. PBMCs were separated using Ficoll-Paque PLUS (GE Healthcare). First, 13 mL of Ficoll-Paque was added to 50 mL centrifuge tubes. Then, 9 mL of blood diluted with 28 mL of PBS was layered on top of it. The suspension was centrifuged at 800 × *g* for 30 minutes after which the PBMC layer was transferred to a new tube. The cells were washed twice, using PBS and centrifugation at 300 × *g* for 15 minutes and at 300 × *g* for 10 minutes. *Ex vivo* PBMCs were conditioned in culture for 1 day and subsequently maintained in culture for 4 days ±5 nM cladribine (optimal cladribine concentration was determined as outlined in the Results section). Each biological replicate was maintained in either duplicate or triplicate for the duration of each experiment. PBMCs were cultured at 37°C in 5% CO_2_, in RPMI 1640 medium (Corning 15-040-CV) containing 25 mM HEPES, and 10 % FBS, supplemented with 1× Glutamax (Gibco), 1 mM sodium pyruvate and penicillin/streptomycin. Cell viability estimates were performed using a standard semi-automated trypan-blue exclusion method using the Countess II (Invitrogen).

### CD4+, CD8+, and CD19+ cell separation and DNA extraction

After maintaining the PBMC cultures for 4 days ±5 nM cladribine, we separated CD4+, CD8+, and CD19+ cells. Since the relative proportions of these cells varies in PBMCs, we used different amounts of the cultured PBMCs to extract each cell population in order to have similar quantities of DNA from each cell population. We used 60% of the PBMCs for separating the CD19+ cells, 25% for CD8+, and 15% for CD4+ cells. We used positive separation with MACS CD4, CD8, and CD19 antibody MicroBeads (Miltenyi Biotec, Bergisch Gladbach, Germany) using an OctoMACS magnetic separator (Miltenyi Biotec) following the manufacturers protocol. DNA was extracted using QIAamp DNA Mini Kit (Qiagen).

### Cell proliferation in T-cell lines

For each cell line, cell viability and proliferation were routinely monitored over short term and extended culture, by trypan blue exclusion and cell counting, and using the WST-1 assay (Sigma–Aldrich), respectively. The data represent *n* = 5 independent biological experiments.

### Immunoblotting in T-cell lines

Immunoblotting was carried out as described [22]. Briefly, cells were solubilized in phosphate buffered saline, 1% dodecyl-maltoside, 1 mM phenylmethylsulfonyl fluoride, and complete protease inhibitor (Thermo Fisher). Equal protein concentrations were separated by Tris-glycine SDS-PAGE and transferred to nitrocellulose by semi-dry transfer. Membranes were blocked in Tris-buffered saline (0.1% Tween 20) (TBST) with 1% milk at room temperature for 1 hour, and then primary antibodies were incubated overnight at +4°C in 5% bovine serum albumin/tris-buffered saline and detected the following day with secondary horseradish peroxidase conjugates (Jackson ImmunoResearch) using enhanced chemiluminescence with film. Primary antibodies from Proteintech Group: uL11m (15543-1-AP, 1:20 000); mS35 (15989-1-AP, 1:5000); MT-ATP6 (55313-1-AP, 1:2000); CANX (10427-2-AP). Primary antibodies from Abcam/Mitosciences: MT-CO1 (1D6E1A8, 1:500) and SDHA (C2061/ab14715, 1:10 000). Primary antibody from Santa Cruz: TOM40 (sc-11414, 1:5000). Primary antibody from Cell Signaling: CASPASE 3 (#9662). Representative data of independent experiments were cropped in Adobe Photoshop with only linear corrections to brightness applied. Independent experiments were performed and quantified.

### Mitochondrial membrane potential determination in T-cell lines

Cells were treated with 200 nM tetramethylrhodamine, methyl ester (TMRM, Life Technologies). For a positive control, cells were treated with the protonophore carbonyl cyanide *m*-chlorophenyl hydrazone (CCCP, 10 μM), which dissipates the membrane potential. After TMRM staining, cells were analyzed by flow cytometry (Accuri C6, Becton Dickinson or a BD LSR II). Independent experiments were performed and quantified.

### Metabolic labeling of mitochondrial protein synthesis in T-cell lines

Mitochondrial protein synthesis was analyzed by metabolic labeling with ^35^S methionine/cysteine, as described [22]. Briefly, cells were pretreated with anisomycin (100 µg/mL) to inhibit cytoplasmic translation then pulsed with 200 µCi/mL ^35^S Met-Cys (EasyTag-Perkin Elmer). Equal amounts of sample protein were first treated with Benzonase (Thermo Fisher) on ice and then resuspended in 1× translation loading buffer (186 mM Tris-hydrochloride pH 6.7, 15% glycerol, 2% SDS, 0.5 mg/mL bromophenol blue, 6% β-mercaptoethanol). A 12–20% gradient Tris-glycine SDS-PAGE was used to separate samples then dried for exposure with a Phosphoscreen and scanned with a Typhoon 9400 or Typhoon FLA 7000 (GE Healthcare) for quantification. Gels were rehydrated in water, and Coomassie stained to confirm loading.

### DNA extraction and mitochondrial DNA copy number in T-cell lines

mtDNA copy number and integrity were determined in cultured leukemic cell lines as described [22]. Briefly, whole cell DNA was isolated with QIAamp DNA Mini Kit (QIAGEN). DNA (5 μg) was digested with PvuII then separated on a 0.8% agarose gel followed by alkaline transfer to Hybond-XL membrane (GE Healthcare). Two oligonucleotide probes for mtDNA (5ʹ-GGCTCCAGGGTGGGAGTAGTTCCCTGC; 5ʹ-CCTCCCGAATCAACCCTGACCCCTCTCC) and three for 18S rDNA (5ʹ-GGCCCGAGGTTATCTAGAGTCACC; 5ʹ-TATTCCTAGCTGCGGTATCCAGGC; 5ʹ-ACCATCCAATCGGTAGTAGCGACG) were 5ʹ end labeled with gamma ^32^P ATP by T4 Polynucleotide Kinase (NEB) and hybridized (50% formamide, 7% SDS, 0.25 M sodium phosphate pH 7.2, 10 mM EDTA pH 8.0, 0.24 M NaCl_2_) overnight at 37°C. Membranes were washed once with 2× SSC/0.1% SDS for 60 minutes, followed by 0.5× SSC/0.1% SDS for 60 minutes and finally in 0.1× SSC/0.1% SDS for 30 minutes. All washings were performed at 37°C. Membranes were dried then exposed to a Phosphoscreen (GE Healthcare) and scanned with a Typhoon 9400 (GE Healthcare).

### Mitochondrial cytochrome-*C* oxidase I mRNA analysis

Total cellular RNA was isolated using Trizol (Invitrogen) according to the manufacturer’s instructions. A cDNA synthesis of the MT-CO1 mRNA was performed with RT-PCR (5ʹ-TGTAAAACGACGGCCAGTTATACCTATTATTCGGCGC; 5ʹ-CAGGAAACAGCTATGACCC GGGTTCTTCGAATGTGTG), followed by amplicon and next-generation sequencing analysis as described [23].

### Next-generation sequencing analysis of mtDNA

Because of nuclear-encoded mitochondrial pseudogenes, we developed an approach to ensure specific sequencing of the mitochondrial genome. Primer pairs developed by Illumina (MTL-F1/MTL-R1 5ʹ-tgtaaaacgacggccagt AAAGCACATACCAAGGCCAC-3ʹ, 5ʹ-caggaaacagctatgacc TTGGCTCTCCTTGCAAAGTT-3ʹ and MTL-F2/MTL-R2 5ʹ-tgtaaaacgacggccagt TATCCGCCATCCCATACATT-3ʹ, 5ʹ-caggaaacagctatgaccAATGTTGAGCCGTAGATGCC-3) were used to amplify 9.1 kb and 11.1 kb overlapping amplicons of the mitochondrial genome using the Kapa HiFi long-range polymerase (KM3005 Roche). The 9.1 kb fragment was amplified from the *ex vivo* cell DNA by Kapa HiFi HotStart ReadyMix (KM2602 Roche). Amplified products were size selected, purified, and then analyzed using the Agilent Fragment Analyser before proceeding with the library generation for next-generation sequencing. All of the 11.1 kb amplicons passed the quality control for library generation. One MS patient (MS1) had heteroplasmy in the 9.1 kb fragment selectively in T cells, and only part of the 9.1 kb amplicon could be sequenced in this case. The mean coverage per site was 86 208× and 85 980× in untreated and treated CCRF cells, respectively. The mean coverage per site was 12 359× and 11 573× in the untreated and treated primary cells. Library preparations and Illumina Sequencing reactions in the T-cell lines were performed by the Max Planck-Genome-centre Cologne, Germany (http://mpgc.mpipz.mpg.de/home/) and in the *ex vivo* lymphocytes at the Biomedicum functional genomics unit at the Helsinki University (https://www2.helsinki.fi/en/infrastructures/genome-analysis/infrastructures/biomedicum-functional-genomics-unit).

### Somatic mutation calling in the T-cell lines

Sequencing reads were trimmed with Flexbar v2.5 [24] for TruSeq adapters and base call quality (default parameters except -q 28 -m 50 -ae ANY -ao 10). Reads containing the M13 tag sequences in forward or reverse orientation were removed by Flexbar (parameters -bo 15 -bt 2 -bu -be ANY) in order to avoid primer containing reads and highly biased coverage at the amplicon edges. Unassigned reads (without the M13 tag) were aligned with BWA v0.7.12-r1039 invoking mem [25] (parameters -T 19 -B 4 -L 5,4) to the human mitochondrial reference sequence (NC_012920.1). Uniquely aligned reads (samtools view -q 1) were converted to bam format, sorted and indexed with samtools [26]. Per base coverage was determined with bedtools v2.22.1 [27] genomecov (parameters -split -d). Variants were detected with Lofreq* v2.1.2 [28] as follows. First, the indel qualities were set by command lofreq indelqual—dindel, then variants were called including indels with minimum base call quality 30 by command lofreq call-parallel --pp-threads 20 -N -B -q 30 -Q 30—call-indels—no-default-filter. Variant results were further filtered for minimum variant quality Phred score 70 and maximum strand bias Phred score 60 (strand bias filter applied only if ≥85% of the variant supporting reads were on a single strand) by command lofreq filter—no-defaults—snvqual-thresh 70—sb-incl-indels -B 60. Next, the variants were filtered for minimum of 15 variant supporting reads using snpSift filter with the expression DP*AF ≥15 and for minimum of three variant supporting reads on each strand (expression DP4[2] ≥3 & DP4[3] ≥3). Finally, only variants and indels present at positions covered by >35 000 reads and minimum variant allele frequency (AF value) of 1% were accepted.

### Somatic mutation calling in primary lymphocytes

After an initial quality control using FastQC [29], sequencing reads were trimmed with Cutadapt 1.18 [30] to remove primer containing reads and to keep a phred quality of above 20. Trimmed, reads were aligned with BWA v0.7.17 invoking mem [25] (parameters -A 1, -B 4, -O 6, -E 1, -k 19, M) to the human mitochondrial reference sequence (NC_012920.1). The MarkDuplicates function from Picard 2.18.23 [31] was used to remove duplicated reads. Prior to variant calling, we perform realignment for insertions and deletions (indels) based on indels in the input reads with minimum of three indel containing reads. VarDict 1.5.8 [32] in its amplicon aware mode was used for subsequent variant calling using the following parameters in the main analysis: minimum allele frequency (AF) 0.01 (default in VarDict), minimum number of variant reads 4, minimum “good” base quality 25, minimum number of reads for strand bias 2, minimum q ratio 1.5, minimum mapping quality 10, maximum number of mismatches in reads 8, and indel size 120. We also performed sensitivity analyses, in which the identified variants were filtered using AF = 0.001, 0.002, 0.005, and 0.025 and by reducing the minimum base quality to 22.5 with at least two high-quality reads and a minimum total depth of 3.

## Statistical analysis

In the T-cell line analysis data represents mean ± standard deviation (SD) of the number (*n*) of independent biological experiments indicated in the figure legend.

We used the Student’s *t*-test analysis to assess the significance of difference between the overall mutation rates in cells from the pool of patients treated with cladribine versus untreated. For all the other analyses of mutation rates, we employed the Wilcoxon rank sum test because it does not require the assumption of normal distribution to be fulfilled. The significance threshold for hypothesis testing, α, was set to 0.05. We did not perform power analysis for the sample size calculation; our study represents an exploratory analysis in 12 subject’s cells (4 controls and 8 MS patients).

## Results

### The inhibitory effect of cladribine on cell proliferation of cultured T-cell lines

**Figure 1.**
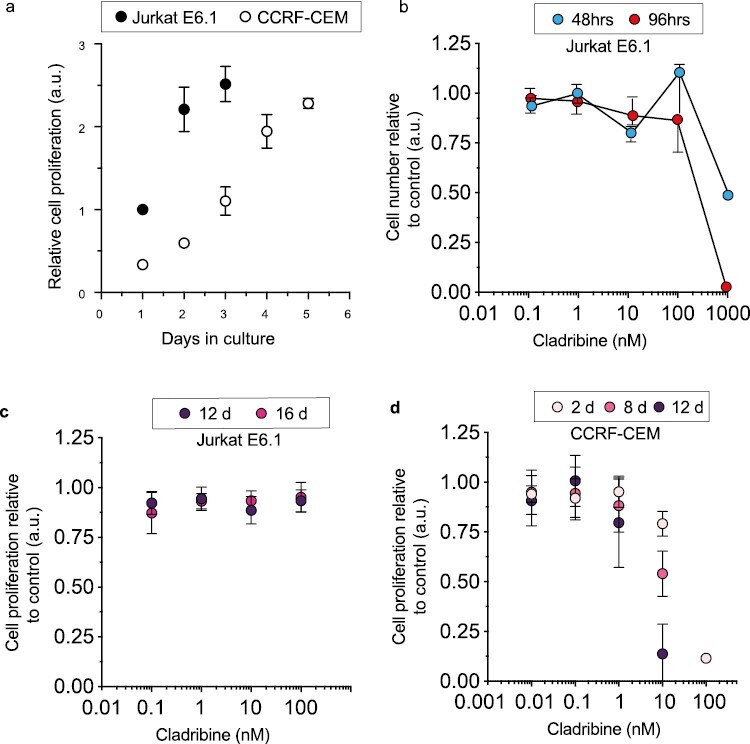
Proliferation rates of the T-cell lines and effects of cladribine on cell viability. (a) The growth rate of Jurkat E6.1 is faster than the CCRF-CEM cell line (*n* = 3). (b) A cladribine dose response curve in Jurkat E6.1 cell line for cell proliferation with 48 hours (*n* = 5) and 96 hours culture (*n* = 5). (c) The effect of cladribine on cell proliferation in Jurkat with 12 days (*n* = 5) and 16 days in culture (*n* = 5). (d) The effect of cladribine on cell proliferation in CCRF-CEM with 2 days (*n* = 5), 8 days (*n* = 4), and 12 days (*n* = 4) in culture (*n* = 5).

Oral administration of a 10 mg cladribine tablet in patients leads to transient peak plasma concentration in the range of 22–29 ng/mL [5] (64–84 nM) [20]. Therefore, we here established a pharmacologically relevant dose response curve for cladribine in two leukemic T-cell lines with distinct cell proliferation profiles, with a short duration of treatment, mimicking the situation in MS patients (Fig. 1a). In the more rapidly proliferating Jurkat E6.1 cell line, concentrations of cladribine up to100 nM had no inhibitory effects on cell proliferation even after extended culture (Fig. 1b). In contrast, the CCRF-CEM cell line that had a slower rate of proliferation (Fig. 1a) was susceptible to cladribine at <100 nM concentrations in a concentration and time-dependent manner (Fig. 1a and c).

### The effect of cladribine on mitochondrial function of cultured T cells

**Figure 2.**
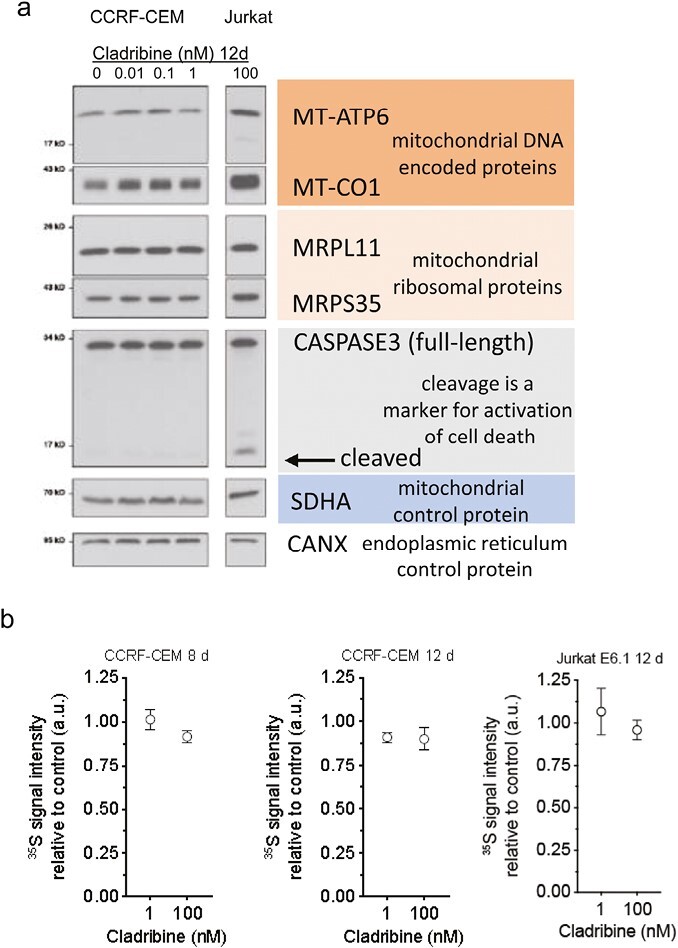
Effect of cladribine on mitochondrial protein synthesis. (a) Immunoblot of MT-ATP6, MT-CO1, MRPL11, MRPS35, CASPASE3, SDHA, and CANX after 12 days culture with cladribine. In this experiment, 10 nM cladribine in CCRF cells resulted in extensive cell death, and immunoblot was not performed. (b) A pulse metabolic labeling of mitochondrial protein synthesis. The effect on mitochondrial protein synthesis in the CCRF-CEM and Jurkat cells after treatment with cladribine for 8 days (*n* = 3) or 12 days (*n* = 3).

**Figure 3.**
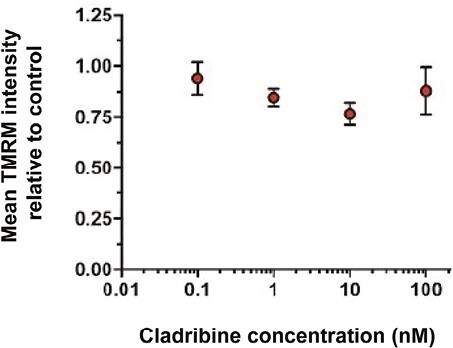
Effect of cladribine on mitochondrial membrane potential in CCRF-CEM cells. Mitochondrial membrane potential at 0.1 nM, 1 nM, 10 nM and 100 nM concentrations of cladribine for 16 days culture (*n* = 3).

At effective concentration of cladribine, we did not detect any alterations in mitochondrial protein synthesis or steady-state protein levels (Fig. 2) in either cell line. The CCRF-CEM cell line appeared susceptible at <100 nM cladribine in a concentration and time-dependent manner (Fig. 1d). Concentrations up to 1 nM did not have any effect on steady-state proteins and/or cleavage of the proapoptotic protease CASPASE3 in CCRF-CEM cells (Fig. 2a). Concentrations up to 100 nM were further tested in 8 and 12 days cultures, but no effect on mitochondrial protein synthesis was found (Fig. 2b). Furthermore, mitochondrial membrane potential in CCRF-CEM cells at 1 nM, 10 nM and 100 nM concentrations in 8 and 12 days culture was not affected by cladribine (Fig. 3). Jurkat cells treated with 100 nM cladribine did neither exhibit any clear effect in mitochondrial protein synthesis (Fig. 2a). Some CASPASE3 cleavage was observed at 100 nM in Jurkat cells (Fig. 2a), although this concentration did not show clear inhibitory effect in the previous proliferation assay (Fig. 1b).

### Cladribine does not affect mtDNA nor mtRNA integrity in T cells

**Figure 4.**
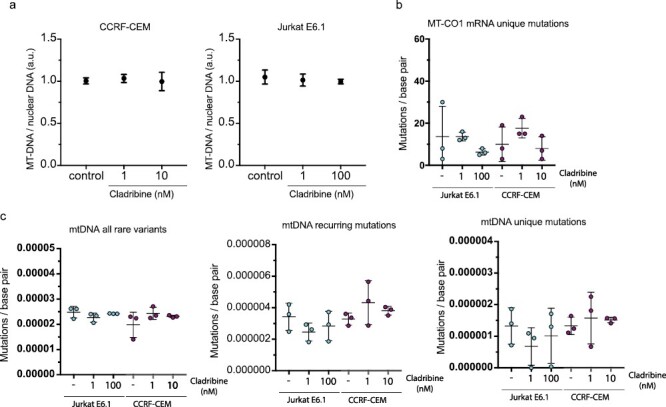
Effects of cladribine on mtDNA copy number and mutagenesis in the T-cell lines. (a) mtDNA copy number after extended culture either with cladribine or without (control). Southern blotting of total DNA extracted from the cells (*n* = 3). Data are expressed as the mtDNA abundance relative to the copy number of the nuclear-encoded 18S rRNA gene a.u. arbitrary units. (b) The mutational effect of cladribine on unique mutation in mRNA of Mitochondrial Cytochrome-*C* Oxidase I (MT-CO1) mRNA (*n* = 3). (c) The mutational effect of cladribine on mtDNA. Mutation abundance for all somatic (rare) variants, recurring mutations, and unique mutations in the mitochondrial genome (*n* = 3).

We next examined the effects of cladribine on mtDNA copy number and integrity, as these play a critical role in determining mitochondrial function. Changes in mtDNA copy number would lead to changes in mitochondrial function. We did not detect any significant differences in the mtDNA copy number between control cells and cells exposed to cladribine even with extended time of exposure (16 days) (Fig. 4a).

We applied next generation sequencing to study mutations in the mtDNA after cladribine exposure. We looked for the abundance of somatic mutations, recurring mutations and for unique mutations in the mitochondrial genome. We did not detect any difference between the control and cladribine-exposed cells on any mutational measure. The level of mutations was not increased due to cladribine in either cell line (Fig. 4b). Since we did not see any effect of cladribine on DNA mutagenesis nor protein synthesis, we concentrated only on the analysis of MT-CO1 mRNA to study the mutational effect of cladribine on mtRNA. We used the same extended culture conditions and concentrations as for the mtDNA analysis. We did not observe any effect of cladribine on unique mutations in MT-CO1 mRNA (Fig. 4c).

### Somatic mutations in mtDNA in human cultured *ex vivo* lymphocytes

We studied the effect of cladribine in primary human lymphocytes, which are less proliferative, and potentially more sensitive than leukemic T-cell lines. Optimization and dose response experiments were done with *ex vivo* PBMCs. At 5 nM cladribine concentration during a 4-day incubation, we observed a modest 10.4% increase in cell death in cladribine-treated versus nontreated cells. Higher concentrations (50 nM) induced fast cell death (acute toxicity), whereas longer incubation times (>5 days) resulted in increased cell death in nontreated cells. Hence, to mimic slow cell depletion in this culture system, 5 nM cladribine for 4 days was selected. These results are in line with previous data indicating that 10 nM cladribine would be too high, because of significant cytotoxicity already after 2 days in cultured *ex vivo* human lymphocytes [17, 33]

**Figure 5.**
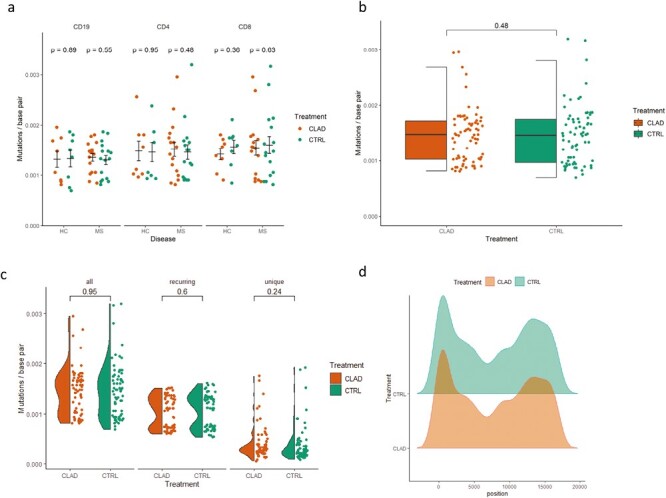
Cladribine has no effect on mtDNA mutagenesis in cultured lymphocytes of 12 donors (8 MS patients and 4 controls). (a) Mutation rate per cell type and disease status. (b) Overall mutation rate, all donors. (c) Mutation rates for recurring and unique mutations. (d) Similar distribution of mutations in mtDNA in treated and nontreated cells. Wilcoxon rank sum test was used to assess the significance of difference between groups in (a) and (c). For (b), the Student’s *t*-test was used.

We obtained blood samples from eight patients with relapsing MS and four controls. CD4+, CD8+, and CD19+ cell populations were enriched from the treated (5 nM) and nontreated PBMC cultures after 4 days of cladribine treatment. We used next-generation amplicon sequencing to screen the mtDNA for mutations. There was no difference in the mutation rate in cladribine treated and nontreated CD4+, CD8+, or CD19+ cells in patients with MS or in healthy controls (Fig. 5a). The overall mutation rate was similar in the pooled treated and nontreated cells (Fig. 5b). We also investigated recurring and unique mutations, and there was no difference between treated and nontreated cells (Fig. 5c). Also, the distribution of mutations along the mtDNA sequence was similar in treated and nontreated cells (Fig. 5d). Sensitivity analyses (see Materials and methods section) using variant filtering AF = 0.001, 0.002, 0.005, and 0.025 did not reveal any differences between patients and controls. Taken together, these results suggest that low-dose cladribine exposure does not increase the number of mtDNA mutations in cultured human lymphocytes.

### mtDNA mutation levels in healthy controls, MS patients, and different cell types

**Figure 6.**
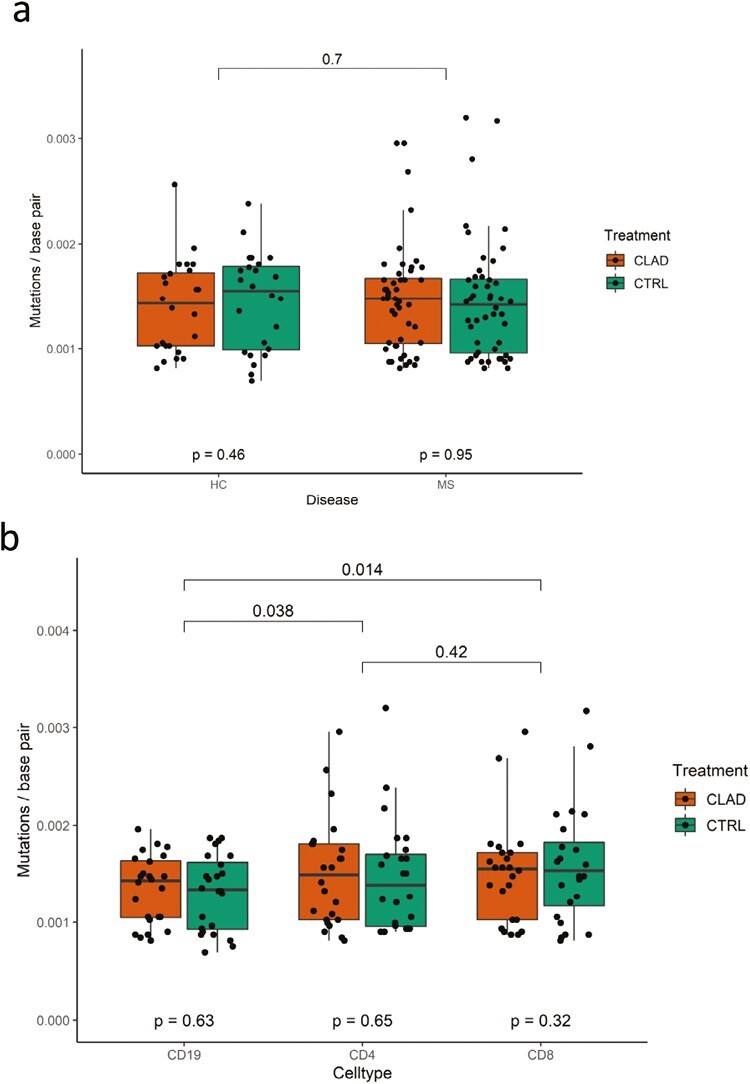
Comparison of mutation rates between multiple sclerosis patients and controls and across lymphocyte subpopulations. (a) Mutation rate is similar in patients with MS versus healthy controls. (b) CD4+ and CD8+ T cells have significantly more mutations than CD19+ B cells. Wilcoxon rank sum test was used to assess the significance of difference between the groups.

Cladribine did not affect the mutation rate in healthy controls (*P* = 0.46) nor in patients with MS (*P* = 0.95), and there was no difference in the overall mutation frequency between MS and controls (*P* = 0.70) (Fig. 6a). When different cell types were studied, we discovered that both CD8+ (*P* = 0.014) and CD4+ (*P* = 0.038) cells had significantly more mutations than CD19+ cells (Fig. 6b). Mutation level in individual donors is shown in Supplementary Fig. S1.

## Discussion

The present study demonstrates that in T-cell lines and in *ex vivo* cultured lymphocytes, cladribine did not affect the integrity of the mitochondrial genome and was not mutagenic to mtDNA. Previous studies with other nucleoside analogs have demonstrated multiple mechanisms of mitochondrial toxicity and cell-specific effects [18]. Our results do not rule out mitochondrial effects, but it is likely that in the studied cell types, cytotoxicity of cladribine is mediated by other mechanisms than mutagenesis of mtDNA.

We were interested in finding out whether cladribine would lead to mtDNA damage in dividing or non-dividing lymphoid cells in concentrations that result in slow cytotoxicity, mimicking the slow depletion of total lymphocytes in patients [7]. The two T-cell lines showed different growth properties and differential growth rates, and sensitivity to cladribine. The slower proliferating CCRF-CEM cell line showed greater sensitivity to cladribine already at lower effective concentrations (10 nM), which resemble the clinical plasma concentrations [12]. Yet, we did not find any increase in mtDNA mutations, mtDNA copy number, or mitochondrial protein synthesis upon cladribine exposure. CASPASE3 cleavage, a marker of apoptosis, was observed only at 100 nM concentrations in Jurkat cells; however, no effects on mtDNA integrity was observed at this concentration. The short duration of treatment (4–16 days in culture) mimics the situation in MS patients, where patients are given yearly two short courses of cladribine tablets treatment for 4–5 days each in the first 2 years of treatment [5] (Mavenclad SmPC). In our culture system, we were able to induce cell depletion without any detectable effects on mitochondrial proteins synthesis or mtDNA integrity. It is likely that similar mechanisms would operate at the concentrations studied here, as these were comparable to those found in MS patients. The next-generation sequencing analyses of the cultured primary human lymphocytes from MS patients or controls, which are mainly non-dividing or slowly proliferating, did not show any increased mutation levels in cladribine-treated cells, and the mutation distribution (Fig. 5d) within mtDNA was remarkably similar in treated and nontreated cells. These results are in line with the results obtained previously in bacteria or mammalian cells, before the next-generation sequencing era, which suggested that cladribine is not mutagenic *in vitro* [5].

A greater decline of CD19+ B-cell counts as compared to CD4+ and CD8+ T-cell counts has been reported after cladribine tablets dosing in MS patients [7]. In our study, the different lymphocyte subpopulations exhibited different levels of somatic mutations (CD8+ and CD4+ levels >CD19+ levels), but the levels were not altered by cladribine. This observation parallels with previous data showing that T cells in blood have more detectable somatic mutations in the nuclear genome than B cells [34, 35]. T cells may generally have a higher somatic mutation rate than B cells, or their clonal development may make these mutations more readily detectable [36]. There was no difference in the overall number of somatic mutations in mtDNA between MS and controls consistent with previous data on nuclear DNA [36, 37]. The *ex vivo* lymphocytes consist of multiple clones that have their own mutational histories. This mutational history of the cells results in a technical challenge (noise), as the hypothetical new mutations occurring during cell culture (signal) should be detected above the noise. Another level of noise is generated during PCR amplification, library preparation, and sequencing. This technical noise limits our sensitivity to detect new mutations, especially in the *ex vivo* lymphocytes. For this reason, to achieve maximal sensitivity, we performed sensitivity analyses with multiple AFs and made separate analyses on unique and recurring mutation (Fig. 4c).

In summary, our work suggests that effective concentrations of cladribine do not alter mitochondrial genome integrity or mtDNA mutation levels in conventional T-cell lines and cultured *ex vivo* lymphocytes. It will be interesting to further characterize the effects of cladribine on additional parameters of cellular homeostasis. Regardless, our data consolidate the previous findings of cladribine not being a mutagenic agent and expand our understanding of its effects in clinically relevant cell types.

## Supplementary Material

uxad112_suppl_Supplementary_Figure_S1

uxad112_suppl_Supplementary_Table_S1

## Data Availability

Data available upon reasonable request.

## Abbreviations:

ADA: adenosine deaminase
EBV: Epstein-Barr virus
MS: multiple sclerosis
mtDNA: mitochondrial DNA
mtRNA: mitochondrial RNA.
