# Supplementary figures and images for: Cultured lymphocytes’ mitochondrial genome integrity is not altered by cladribine
Source: Clin Exp Immunol. 2023 Oct 20;214(3):304–13. doi: 10.1093/cei/uxad112 (PMC10719213; doi:10.1093/cei/uxad112)

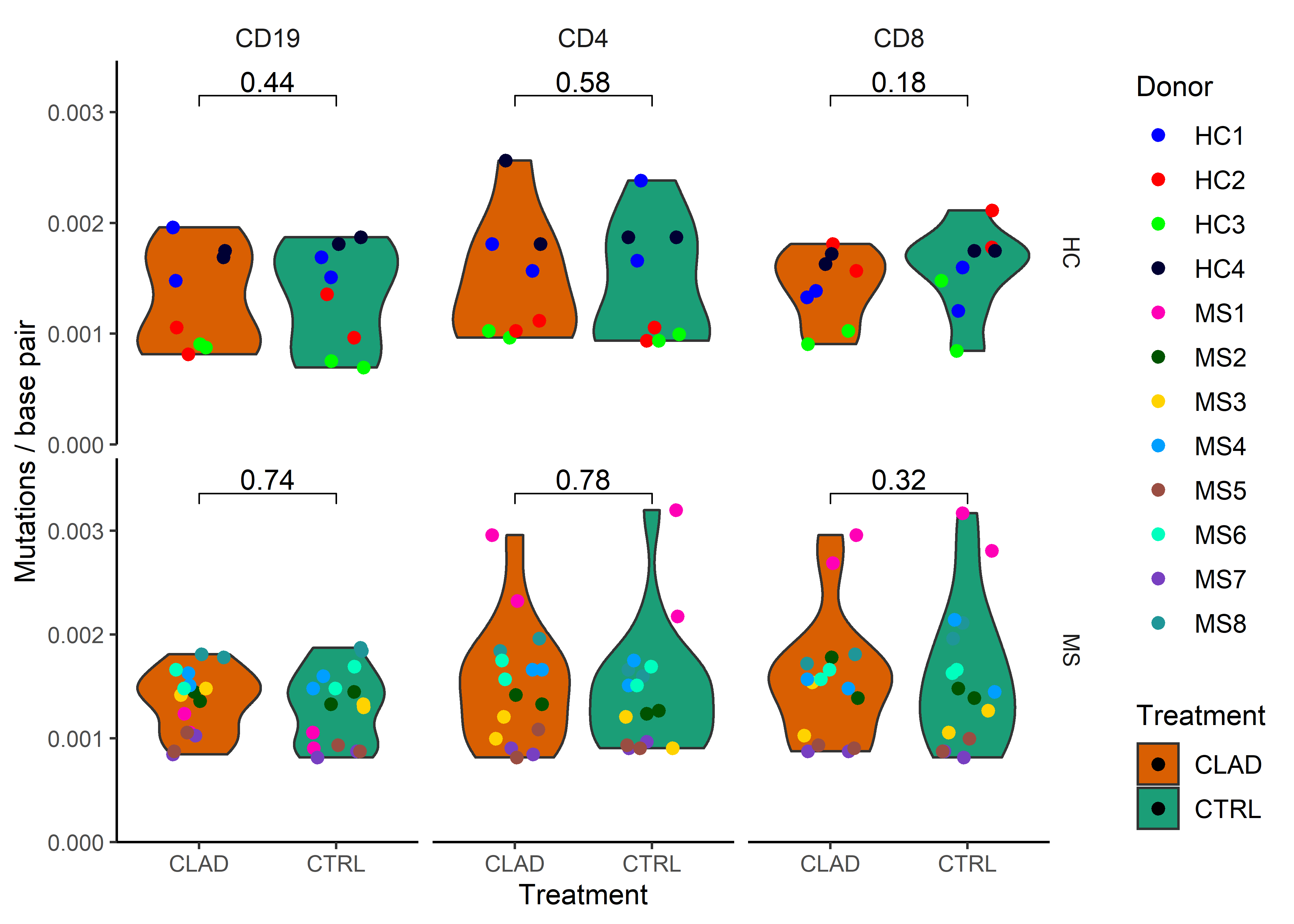


Supplementary Figure 1. Mutation level in individual donors

Supplement: uxad112_suppl_Supplementary_Figure_S1 [file uxad112_suppl_supplementary_figure_s1.docx]
